# Verapamil Blocks Scopolamine Enhancement Effect on Memory Consolidation in Passive Avoidance Task in Rats

**DOI:** 10.3389/fphar.2017.00566

**Published:** 2017-08-23

**Authors:** Verónica Giménez De Béjar, María Caballero Bleda, Natalija Popović, Miroljub Popović

**Affiliations:** ^1^Department of Neurology, Hospital Quirónsalud Murcia Murcia, Spain; ^2^Instituto Murciano de Investigación Biosanitaria Virgen de la Arrixaca Murcia, Spain; ^3^Department of Human Anatomy and Psychobiology, Faculty of Medicine, University of Murcia Murcia, Spain

**Keywords:** memory consolidation, passive avoidance, verapamil, scopolamine, rat

## Abstract

Our recent data have indicated that scopolamine, a non-selective muscarinic receptor antagonist, improves memory consolidation, in a passive avoidance task, tested in rats. It has been found that verapamil, a phenylalkylamine class of the L-type voltage-dependent calcium channel antagonist, inhibits [3H] *N*-methyl scopolamine binding to M1 muscarinic receptors. However, there are no data about the effect of verapamil on memory consolidation in the passive avoidance task, in rats. The purpose of the present study was to examine the effects of verapamil (0.5, 1.0, 2.5, 5.0, 10, or 20 mg/kg i.p.) as well as the interaction between scopolamine and verapamil on memory consolidation in the step-through passive avoidance task, in Wistar rats. Our results showed that verapamil (1.0 and 2.5 mg/kg) administered immediately after the acquisition task significantly increased the latency of the passive avoidance response, on the 48 h retested trial, improving memory consolidation. On the other hand, verapamil in a dose of 5 mg/kg, that *per se* does not affect memory consolidation, significantly reversed the memory consolidation improvement induced by scopolamine (1 mg/kg, i.p., administered immediately after verapamil treatment) but did not change the passive avoidance response in rats treated by an ineffective dose of scopolamine (30 mg/kg). In conclusion, the present data suggest that (1) the post-training administration of verapamil, dose-dependently, improves the passive avoidance response; (2) verapamil, in ineffective dose, abolished the improvement of memory consolidation effect of scopolamine; and (3) exists interaction between cholinergic muscarinic receptors and calcium homeostasis-related mechanisms in the consolidation of emotional memory.

## Introduction

The influx of Ca^2++^ through the L-type voltage-gated calcium channels (LVGCCs) promotes several molecular processes that are engaged in learning and memory (Singewald et al., 2015; Bas-Orth et al., 2016; Sachser et al., 2016; Michalak and Biala, 2017; Wiera et al., 2017). Age-related memory loss as well as memory impairment in neurodegenerative and psychiatric diseases has been related to the LVGCCs dysfunction with consequent dysregulation of calcium homeostasis (Barad, 2003; Thibault et al., 2007; Berger and Bartsch, 2014; Yoshimizu et al., 2015; Zanos et al., 2015).

Initially, LVGCCs antagonists, such as benzothiazapines (e.g., diltiazem); dihydropyridines (e.g., amlodipine, felodipine, isradipine, nicardipine, nifedipine, nimodipine, and nisoldipine), diphenylalkylamines (e.g., flunarizine), and phenylalkylamines (e.g., verapamil), were defined as vasodilator, antiarrhythmic, and antianginal agents (Godfraind, 2017). Nowadays, LVGCCs are recognized for the treatment of central nervous system disorders like bipolar disorder (Cipriani et al., 2016), epilepsy (Nicita et al., 2016), and headache (Tfelt-Hansen and Tfelt-Hansen, 2009) with perspective to be also used for the treatment of Parkinson’s disease (Stayte and Vissel, 2014; Surmeier et al., 2017), mood disorders (Kabir et al., 2017), and dementia (Nimmrich and Eckert, 2013).

In comparison to other LVGCCs antagonists, verapamil effects on memory formation are highly inconsistent. Its effect is not only dependent on dose, duration of the treatment, and memory trace phase, but also on memory task performance and species. In mice, acute verapamil treatment improves acquisition in passive avoidance and elevated plus maze tasks (Biala et al., 2013; Michalak and Biala, 2017) while does not affect acquisition in conditioned avoidance response, T-maze, and linear maze tasks (Malekar et al., 1999; Quartermain and Garcia de Soria, 2001; Quartermain et al., 2001). It has been found that acute verapamil treatment impairs familiarity discrimination and perirhinal plasticity, in rats (Seoane et al., 2009). Rats exposed for prolonged period to a high dose of verapamil (50 mg/kg) impaired passive avoidance learning, while lower doses applied either acutely or chronically did not affect passive avoidance performance (Lazarova-Bakarova et al., 1997; Lashgari et al., 2006). The chronic verapamil treatment in rats does not modify acquisition but prejudices retention of the radial maze task (Borroni et al., 2000; Woodside et al., 2004).

Memory consolidation is a fragile part of memory traces that occurs immediately after a learning event and lasts for several hours. In this period, the synthesis of plasticity-related proteins and consequent changes in synaptic strength bring to long-term memory storage in the brain (McGaugh, 2000; Kandel, 2001). Previous studies based on pharmacological modulation of cholinergic transmission by scopolamine, non-selective antimuscarinic agent, reported that scopolamine either does not interrupt (Elrod and Buccafusco, 1988; Cruz-Morales et al., 1992; Roldán et al., 1997; Mishima et al., 2001), impairs (Doyle and Regan, 1993; Sigala et al., 1997; Murphy et al., 2001; Hefco et al., 2003; Foley et al., 2004; Gutierres et al., 2012; Popović et al., 2015), or even improves memory consolidation (Popović et al., 2015), in rats tested in the passive avoidance task. The improving effect was only obtained if scopolamine was administered within 6.5 h after learning and when animals were tested 48 h after acquisition (Popović et al., 2015). Systemic post-training treatment with verapamil impairs habituation in rats exposed to the open filed task (Popović et al., 2016), but in mice preserves memory consolidation in the passive avoidance (Quartermain et al., 1993; Masoudian et al., 2015) and improves retention in the linear maze and elevated plus maze tasks (Biala et al., 2013).

It has been suggested by Quartermain et al. (2001) that the lack of consistency in verapamil effects on memory formation could be due to its number of side effects. Verapamil shares a vasodilatory effect with other LVGCCs antagonists, but together with diltiazem expresses depressant effect on heart rate (van Zwieten and Pfaffendorf, 1993; Noll et al., 1998). Moreover, verapamil tends to suppress the activity of sympathetic nervous system (Noll et al., 1998). Besides, verapamil does not block only LVGCCs channels (being Cav1.2 channels more sensitive than the Cav1.3 ones), but also Cav2.1, Cav2.2, Cav2.3, and Cav3.2 channels too (Ishibashi et al., 1995; Cai et al., 1997; Dobrev et al., 1999; Tarabova et al., 2007; Kuryshev et al., 2014). It has been demonstrated that in several brain regions (e.g., cerebral cortex, hippocampus, and hypothalamus), verapamil acts as an antagonist of muscarinic (Baumgold, 1986; Popova et al., 1990), serotoninergic (Taylor and Defeudis, 1984; Adachi and Shoji, 1986; Green et al., 1990; Popova et al., 1991; Shad and Saeed, 2007), dopaminergic (Sitges and Guarneros, 1998), α- and β-adrenergic (Galzin and Langer, 1983; Staneva-Stoytcheva et al., 1990, 1992), and GABAergic receptors (Staneva-Stoytcheva et al., 1991). In contrast to dose-dependent increase of serotonin and dopamine release, verapamil completely abolishes norepinephrine release in rat hippocampal synaptosomes (Sitges and Reyes, 1995). Aside from these actions on neurotransmission, verapamil is a standard P-glycoprotein inhibitor (Bendayan et al., 2002) and small conductance calcium-activated potassium channels (SK channel) antagonist (Tao et al., 2013).

The relationship between calcium homeostasis, cholinergic system activation and learning and memory, remains to be completely elucidated. Our previous studies indicated that acute and chronic verapamil treatment could ameliorate morphological, physiological, cognitive, and non-cognitive behavioral dysfunctions in rats with cholinergic depletion, suggesting possible relations between cholinergic function and calcium metabolism (Popović et al., 1997a,b,c, 1998a,b, 1999, 2006; Caballero-Bleda et al., 2001). Similarly, Sekhar et al. (2016) demonstrated that verapamil improves scopolamine-induced memory impairment in elevated plus maze and novel object recognition tests. Although evidence exists that verapamil, at the subthreshold, ineffective dose, significantly blocks the improving effect of nicotine on memory consolidation in the elevated plus maze task (Biala et al., 2013), there are no data whether verapamil can modify scopolamine effects on memory consolidation. The aim of the present study was to evaluate the effect of verapamil as well as the interaction between cholinergic muscarinic receptors and calcium homeostasis on memory consolidation, in the passive avoidance task, in rats.

## Materials and Methods

### Experimental Animals

Experiments were carried out on male Wistar rats (200–250 g). The animals were housed in standard Makrolon cages on sawdust bedding. They were kept in an air-conditioned room (20 ± 1°C), at 30% humidity, and under a 12 h light/12 h dark cycle (lights on from 08:00 to 20:00 h). Food and tap water were available *ad libitum.* Before the passive avoidance performance, each rat was handled daily for 5 min during 1 week. The handling and the passive avoidance test were performed between 16:00 h and 20:00 h.

The animal maintenance and experiments were performed in accordance with the European Communities Council Directive of 24 November 1986 (86/609/EEC) and the guidelines issued by the Spanish Ministry of Agriculture, Fishing and Feeding (Royal Decree 1201/2005 of 21 October 2005). All procedures with animals were approved by the Animal Ethics Committee of the University of Murcia. Efforts were made to minimize animal suffering and the number of animals used.

### Drugs

Verapamil and scopolamine hydrobromide were provided by Sigma, St. Louis, MO, United States. Saline solutions of verapamil (0.5, 1, 2.5, 5, 10, or 20 mg/kg), scopolamine hydrobromide (1 mg/kg and 30 mg/kg), or their combination (5 mg/kg of verapamil followed by 1 mg/kg of scopolamine) were administered intraperitoneally. Control animals were treated intraperitoneally with physiological saline at the dose of 1 ml/kg body weight.

### Passive Avoidance Test

The passive avoidance testing was done in an automatically operated commercial Passive Avoidance Apparatus (step-through cage 7550; Ugo Basile, Comerio, Italy). The passive avoidance step-through cage was divided into two equal size compartments (insight dimension 22 cm long × 21 cm wide × 22 cm high, each): START (white and illuminated by a 24 V–10 W bulb) and ESCAPE (black and dark). The two compartments are divided by a partition which embodies an automatically operated sliding door at the floor level. On day 1, each rat was exposed to the exploration trial, by placing it in the START chamber (door closed and shock disconnected) and allowed to explore it for 100 s. After that the door was opened, the rat was allowed to enter into the ESCAPE chamber, and when all four paws were in, the automated slide door was closed. The maximum latency to pass from the START to the ESCAPE compartment was set to 60 s. After 10 s, the rat was removed from the ESCAPE compartment and returned to its home cage. On day 2 (acquisition trial), when the rat entered into the ESCAPE compartment, the door was closed and a 1.0 mA shock was delivered for 5 s. Ten seconds later on, the rat was removed from the ESCAPE compartment, drug administered (saline, verapamil, scopolamine, or verapamil followed by scopolamine treatment), and returned to its home cage. According to the latency period to enter into the ESCAPE compartment on day 1 and sensitivity to the shock (vocalization and jumping response) on day 2, the animals were assigned into 11 groups; thus, there were no significant differences between groups. Eight animals were assigned in each tested group. Forty-eight hours after the acquisition trial, the retention trial was carried out. The test was performed in a similar way to the acquisition trial, but no shock was given. The cage catch pan, grid floor, and side walls were cleaned with 70% ethanol before each animal was tested. The most often cut-off times used in the passive avoidance test are 180, 300, and 600 s. In the present study, the cut-off time for the entrance of the rat into the dark compartment was 9 min (maximum time allowed by the used apparatus). The longer cut-off time is when the individual differences become more apparent (Sahgal, 1993). Therefore, for better drug effect discrimination, three cut-off times (180, 300, and 540 s) were analyzed for each animal and group.

### Statistical Analysis

The statistical analysis was made using the SPSS 19.0 statistical package. The data are presented as mean ± standard error of the mean (SEM). The effects of verapamil or scopolamine on the step-through latency were analyzed with the one-way ANOVA, followed by the least significant difference (LSD) *post hoc* test. The two-tailed Student’s *t*-test for independent samples was used to analyze the effect of combined verapamil and scopolamine treatments. Differences were considered statistically significant if *p* < 0.05.

## Results

**FIGURE 1:**
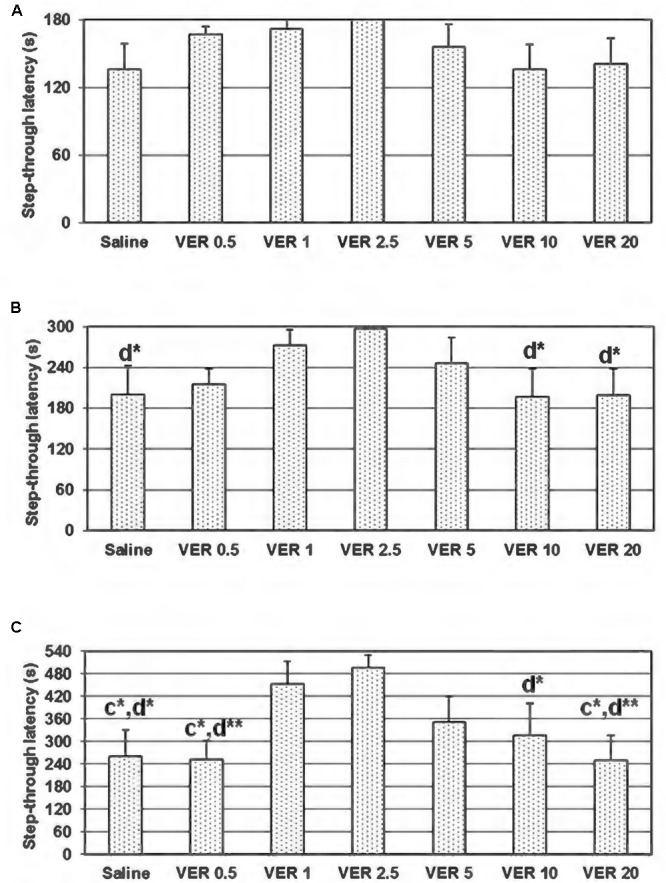
Effect of 0.5, 1, 2.5, 5, 10, and 20 mg/kg of verapamil (VER-0.5, VER-1, VER-2.5, VER-5, VER-10, and VER-20, respectively), administered i.p., immediately after the acquisition trial, on the 48 h retention trial, in the passive avoidance task, in conditions of 180 **(A)**, 300 **(B)**, and 540 s **(C)** cut-off latency. The data are presented as mean ± standard error of the mean (SEM). C-compared to VER-1 and d-compared to VER 2.5; ^∗^*p* < 0.05, ^∗∗^*p* < 0.01.

On the 48 h retention trial of the passive avoidance test, using a maximum cut-off time of 540 s, the one-way ANOVA test showed significant dose-dependent effect of verapamil on the step-through latency (*F*_(6,49)_ = 2,483, *p* = 0.036; **Figure 1C**). In animals treated with verapamil at the doses of 1 and 2.5 mg/kg, the step-through latency on the 48 h retention trial was significantly higher in comparison to the groups treated with saline (*p* = 0.037 and *p* = 0.012, respectively), verapamil in the dose of 0.5 (*p* = 0.029 and *p* = 0.009, respectively) and 20 mg/kg (*p* = 0.028 and *p* = 0.009, respectively). Moreover, animals treated with verapamil at the dose of 2.5 mg/kg showed higher step-through latency in comparison to the animals treated with verapamil at the dose of 10 mg/kg (*p* = 0.05). Although employing a 300 s cut-off time, the one-way ANOVA test showed no significant dose-dependent effect of verapamil on the step-through latency (*F*_(6,49)_ = 1,512, *p* = 0.194), there were significant differences between animals treated with verapamil in the dose of 2.5 mg/kg and animals treated with saline (*p* = 0.043), verapamil in the doses of 10 and 20 mg/kg (*p* = 0.034 and *p* = 0.040, respectively; **Figure 1B**). Applying a 180 s cut-off time, the one-way ANOVA test showed no significant dose-dependent effect of verapamil on the step-through latency (*F*_(6,49)_ = 1,132, *p* = 0.358; **Figure 1A**).

**FIGURE 2:**
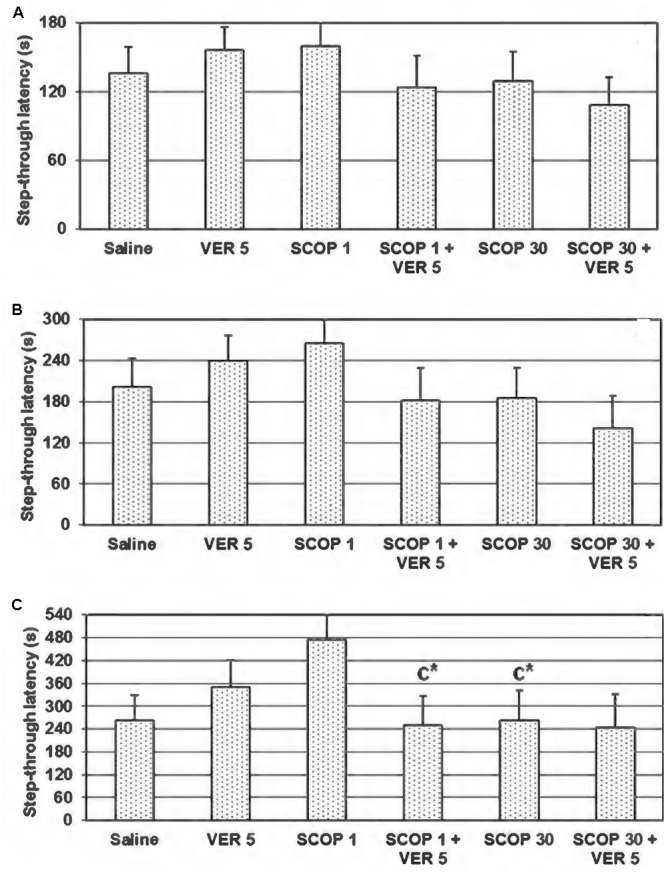
Effect of combined verapamil (VER) 5 mg/kg and scopolamine (SCOP) (1 or 30 mg/kg), administered i.p., immediately after the acquisition trial, on the 48 h retention trial, in the passive avoidance task in conditions of 180 **(A)**, 300 **(B)**, and 540 s **(C)** cut-off latency. Data are presented as mean ± SEM. C-compared to SCOP-1; ^∗^*p* < 0.05.

The one-way ANOVA test did not showed significant dose-dependent effect of scopolamine on the step-through latency, utilizing 180, 300, and 540 s cut-off time (*F*_(2,21)_ = 0.472, *p* = 0.630; *F*_(2,21)_ = 1,074, *p* = 0.360; and *F*_(2,21)_ = 2.995, *p* = 0.72, respectively) (**Figures 2A–C**). However, when evaluating at 540 s cut-off time, the step-through latency was significantly higher in animals treated with scopolamine in the dose of 1 mg/kg, in comparison to those treated with saline and scopolamine in the dose of 30 mg/kg (*p* = 0.045 and *p* = 0.047, respectively; **Figure 2C**). The animals treated by the combined treatment of scopolamine (1 mg/kg) and verapamil (5 mg/kg) significantly reduced the step-through latency in comparison to the group treated only by scopolamine (1 mg/kg) (*t* = 2.219, *df* = 14, *p* = 0.044; **Figure 2C**).

## Discussion

It has been demonstrated that verapamil treatment in the dose of 20 mg/kg does not modify, but that in the dose range from 1 to 10 mg/kg significantly improves consolidation of spatial memory in the linear maze task, in mice (Quartermain et al., 2001). In the elevated plus maze task, only mid-doses used of verapamil (5 and 10 mg/kg) display enhancement effect on spatial memory consolidation, in mice (Biala et al., 2013). The present data indicated that verapamil at the doses of 1 and 2.5 mg/kg, but not at the doses of 0.5, 5, 10, and 20 mg/kg, improved memory consolidation of rats tested in passive avoidance task, confirming the inverted U-shape dose–response curve of its action on memory consolidation. In contrast to the present study, Quartermain et al. (2001) and Masoudian et al. (2015) found that the post-training treatment with verapamil (1, 2, 10, and 20 mg/kg and 1, 2.5, 5, 10, and 20 mg/kg, respectively) did not change the retention of the passive avoidance task in mice. The discrepancies between data obtained in passive avoidance studies could be attributed to the species utilized (mice vs. rats), shock intensity (0.1 or 0.5 vs. 1 mA), duration of the shock (1 or 2 vs. 5 s), acquisition–retention interval (24 or 48 h), as well as the duration of the retention trial applied (400 vs. 540 s).

Glick and Zimmerberg (1971) and Rush (1988) showed that scopolamine in higher dose (10–30 mg/kg) impaired memory consolidation in mice tested in the passive avoidance task. The same authors demonstrated that the dose-dependent impairment is related to the duration of the cut-off latency (180, 300, or 600 s), such as longer cut-off latency and lower scopolamine dose are necessary to induce memory impairment. To discard the influence of the cut-off latency time, in the present study we also performed statistical analyses with two shorter frequently used cut-off latency periods (180 and 300 s). Our data suggest that verapamil administered in the dose of 2.5 mg/kg significantly improved memory consolidation in the passive avoidance task, when 300 and 540 s cut-off time were utilized. In contrast, scopolamine in the dose of 1 mg/kg improved memory consolidation when 540 s (Popović et al., 2015), but not when shorter (180 and 300 s) cut-off time was employed. On the other hand, 30 mg/kg of scopolamine (independent of the cut-off latency time) applied in the present study, as well as 50 mg/kg of it, used by Anagnostaras et al. (1999), did not modify memory consolidation in rats tested in the passive avoidance and fear condition tasks, respectively. These results imply that optimal level of cholinergic neuronal firing, crucial for memory formation, is not only task (Hasselmo and McGaughy, 2004; Hasselmo and Sarter, 2011) but could be also species depending.

The mechanism by which verapamil facilitates or impairs consolidation of emotional memory is still unclear. Findings obtained in Cav1.2 knockout mice argue against the role of the Cav1.2 channels in the consolidation of emotional memory (McKinney et al., 2008). On the other hand, impaired ability of Cav1.3 knockout mice to consolidate contextually conditioned fear but with preserved consolidation in the hidden platform version of the Morris water maze test reveals that the deficits observed in these mice are the result of a disruption of neuronal function within the amygdala (McKinney and Murphy, 2006). Given an inverted U-shape dose–response curve of the verapamil action, Biala et al. (2013) proposed that its action could be due to modulation rather than a complete blockade of LVGCCs (Biala et al., 2013). The facts that: (1) CaV1.2 subtypes are blocked at much lower doses of verapamil in comparison with the CaV1.3 subtypes (Tarabova et al., 2007); (2) higher doses of LVGCCs blockers are required to effectively inhibit brain CaV1.2 and CaV1.3 channels (Helton et al., 2005; Zamponi et al., 2015); and (3) hypotension could improve memory consolidation in the passive avoidance task (Haile et al., 2012), suggest the possibility that outcomes of low doses verapamil treatment, on the passive avoidance consolidation, could be attributed to its effect on the cardiovascular system rather than to the effect on neurons and most likely modulated via CaV1.2 subtypes of LVGCCs.

In view of the facts that verapamil can block SK channel (Tao et al., 2013) and that the blockade of SK2 channels, immediately after the training, enhanced contextual fear memory (Murthy et al., 2015), it could be expected that the effect of verapamil is partially due to the action on these channels, too. On the other side, verapamil expresses both α_1_ and α_2_ adrenergic receptor blocking activity (Müller and Noack, 1988) and blocking of the α_2_ adrenergic receptor subtype improves memory consolidation in the passive avoidance task (Ferry et al., 2015).

Other potential mechanism of verapamil effects on memory consolidation could be related to its antagonistic action on cholinergic receptors. Biala et al. (2013) demonstrated that verapamil, at the subthreshold, ineffective dose (2.5 mg/kg), significantly blocked the improving effect of nicotine (0.035 mg/kg) on memory consolidation, in the elevated plus maze task. Similarly, the present data showed that verapamil, in ineffective dose (5 mg/kg), abolished the improvement of memory consolidation effect of 1 mg/kg of scopolamine but did not change the passive avoidance response when ineffective dose of scopolamine (30 mg/kg) was used. Considering that nicotinic antagonists can block the scopolamine effect of memory formation (Newman and Gold, 2016) and that verapamil inhibited [^3^H] *N*-methyl scopolamine binding to M1 muscarinic receptors in the rat brain cortex (Baumgold, 1986), further studies are need to determine the cascade of verapamil action on memory consolidation.

## Conclusion

As far as we know, the present data represent the first demonstration that verapamil dose-dependently improves memory consolidation of the passive avoidance task in rats, and that exists interaction between cholinergic muscarinic receptors and calcium homeostasis, on memory consolidation.

## Author Contributions

VG, MC, NP, and MP contributed to the design of the study, wrote the protocol, and managed the literature searches; VG, NP, and MP performed the experiments and undertook the statistical analysis; and VG, MC, NP, and MP contributed to drafting the work and have approved the final manuscript.

## Conflict of Interest Statement

The authors declare that the research was conducted in the absence of any commercial or financial relationships that could be construed as a potential conflict of interest.
